# The Impact of Post Activation Potentiation on Achilles Tendon Stiffness, Elasticity and Thickness among Basketball Players

**DOI:** 10.3390/sports6040117

**Published:** 2018-10-12

**Authors:** Beata Pożarowszczyk, Artur Gołaś, Aiguo Chen, Adam Zając, Adam Kawczyński

**Affiliations:** 1 Department of Paralympics Sports, University School of Physical Education, 51-612 Wrocław, Poland; kawczynski.a@gmail.com; 2 Department of Sports Training, The Jerzy Kukuczka Academy School of Physical Education, 40-001 Katowice, Poland; a.golas@awf.katowice.pl (A.G.); a.zajac@awf.katowice.pl (A.Z.); 3 College of Physical Education, Yangzhou University, Yangzhou 225009, China, agchen@yzu.edu.cn

**Keywords:** PAP, tendon viscoelastic properties, myotonometry

## Abstract

The purpose of this study is to examine and further understand the effects of post activation potentiation on Achilles tendon (AT) thickness, elasticity and stiffness among basketball players. Basketball is one of the world’s most popular and widely viewed sports. One of the main factors which athletes depend on during their performance is elastic energy coming straight from the AT. Contractile activity increases the muscular force and is known in science as post activation potentiation (PAP). Twelve basketball players (aged 21.3 ± 2.1 years) from the first Polish league took part in this study. The PAP session consisted of single repetitions of the squat with loads corresponding to 60%, 70%, 80%, 90% and 100% of 1 repetition maximum (RM). The measurement method for AT thickness was ultrasonography and for the elasticity and stiffness was myotonometry. The measurements were taken before and immediately after PAP training session. Obtained results: AT stiffness increased significantly from the baseline post exercise, while AT thickness and elasticity decreased after the physical effort. The exercise in PAP caused significant changes in stiffness, elasticity and thickness of the AT.

## 1. Introduction

Basketball is one of the world’s most popular and widely viewed sports. It is a very dynamic, limited-contact sport with many individual techniques that demonstrate skills such as ball handling, dunking, passing, dribbling, shot-blocking, shooting and rebounding. Thus, basketball requires a challenging combination of agility, speed, technical skill, and strength.

Ankle injuries are a common problem in basketball, including Achilles tendon (AT) structure [1,2]. Although the main cause of injuries in basketball relates to the lateral ankle ligaments the AT plays an important part in basketball-specific movements. Risk factors associated with Achilles injuries include age, changes in player performance characteristics, altered foot or ankle biomechanics, and anatomic variations. AT injuries occur when significant forces are translated through a misaligned tendon with a dorsiflexed foot and an extended knee [3].

AT enables explosive movements by storing and returning elastic Energy [4,5]. Explosive performances in athletes need the elastic energy that AT provides to work properly [6].

Post activation potentiation (PAP) is a phenomenon in which muscular force is acutely enhanced as a result of prior contractile activity. The net augmentation is dependent on the intensity of the preceding conditioning contraction, influencing calcium release and phosphorylation of the regulatory myosin light chain [7]. The mechanisms of PAP are also suggested to relate to enhanced motor neuron recruitment [8], a more favorable central input to the motor neuron, or a contribution from all of these mechanisms [9,10].

Many previous studies examining the PAP phenomenon have employed heavy (75–95% with one repeat maximum) isotonic resistance exercises as the preload stimulus, with some authors also supporting the use of maximal isometric conditioning contractions [11]. The PAP effect depends on the balance between fatigue and neuromuscular potentiation [12], which in turn depends on the type of exercise, volume, intensity, and recovery time [13].

One of the methods that become popular in estimating viscoelastic muscle properties is myotonometry. This easy-to-use technique relies on the recording of natural oscillation of myofascial tissue which is induced by a light mechanical impulse and applied to the muscle in a relaxed state [14]. MyotonPRO is a reliable, valid, responsive and non-invasive device to quantify mechanical properties such as muscle stiffness, tone or elasticity [15].

Stiffness is dependent on the muscle structure (length and cross-sectional area), forces applied, and intrinsic material properties of muscle [16]. The majority of studies examining the muscle biomechanical properties suggested that higher stiffness is beneficial for fast stretch shorten cycle activities and activities involving high movement velocity [17,18,19,20]. Stiffness may also have significant implications in force production within muscles [21].

The purpose of this study was to examine and further understand the effects of post activation potentiation on AT thickness, elasticity and stiffness among basketball players.

## 2. Materials and Methods

### 2.1. Participants

Subjects (n = 12) participating in this study were male basketball players from the first Polish league (aged 21.3 ± 2.1 years; height 194.3 ± 3.3 cm; body mass 82.7 ± 5.8 kg). The training experience of the whole group was on average 9 ± 3 years. Each participant provided informed agreement. All subjects maintained a normal training schedule for 7 days prior to the experiment. All subjects had no AT injury history.

### 2.2. Measurements

#### 2.2.1. Myotonometry

Handheld MyotonPRO device (MyotonPRO, Myoton Ltd., Tallinn, Estonia) was applied to measure AT stiffness in basketball players. Muscle stiffness (St; N/m) characterizes the resistance of soft tissue to a contraction or external force and the ability to restore its initial shape. Five myotonometric measurements were performed at each tested point. During stiffness measurement subjects were lying in a horizontal position on comfortable massage tables. Subjects were instructed to fully relax their muscles during the measurements. Measurements were taken before and immediately after the PAP session. The calculation formula for muscle stiffness is:

(1) $$S=\frac{a_{max}\times m_{probe}}{\Delta l},$$

where *a* is the acceleration of the damped oscillation, Δ*l* is the maximal displacement of the tissue, and *m_probe_* is the mass of the measurement mechanism (MyotonPRO, Myoton Ltd., Tallinn, Estonia).

Elasticity, characterized by logarithmic decrement of the dampened oscillations, is expressed in arbitrary units.

(2) $$\theta =\ln\left(\frac{a_{1}}{a_{3}}\right),$$

This indicates how much mechanical energy is lost in the tissue during an oscillation cycle. The smaller the decrement value, the smaller will be the dissipation of mechanical energy and higher elasticity of a tissue [22]. A decrement value of zero would represent absolute elasticity and zero dissipation of mechanical energy.

#### 2.2.2. Ultrasonography

Subjects were instructed to lie prone with both ankles kept dorsiflexed at a 90-degree angle. The AT of the dominant lower extremity was then observed in the longitudinal axis (sagittal plane). Measurements were conducted with software embedded in the ultrasound scanner and saved on the hard drive of the unit. The measurement of AT thickness was taken at 10 mm proximal to the attachment of the tendon to the reference point of the calcaneal insertion. The reference point was defined proximally as the first hyperechoic region of the calcaneal insertion. Measurements were taken before and immediately after the PAP session.

#### 2.2.3. PAP Procedures

The general warm up included pedaling on a clo-ergometer at a heart rate of 120–130 beats/min, followed by 5 min of dynamic stretching. The specific warm up consisted of three back squats with a load that allowed the participants to complete 20, 15 and 10 repetitions.

During the PAP session the participants performed single repetitions of the squat with loads corresponding to 60%, 70%, 80%, 90% and 100% of 1 RM until their 1 RM was reached [23].

### 2.3. Data Analysis

SPSS 18 statistical software (SPSS Inc., Chicago, MI, USA) was used for data analysis. A two-way ANOVA with repeated measures was used to compare the measurements before and after PAP.

An a-priori power analysis was performed to determine the required sample size using G*Power software (version 3.1.9.2; Kiel University, Kiel, Germany) [24]. For the Achilles stiffness, elasticity and tendon thickness measures, a sample size of 10 subjects per group was deemed adequate to provide 90% power and an alpha level of 0.05 [24].

Intraclass correlation coefficient (ICC) 3.2 was used to determine the intra-rater reliability of the AT thickness variable. Measurement error was calculated with the standard error of measure (SEM), and minimal detectable change (MDC90) represents the error when a measure is taken twice (change over time) [24].

## 3. Results

AT stiffness increased significantly from baseline (727.2 ± 111.57 N/m) to post activation conditions (822.50 ± 119.55 N/m) (*p* < 0.001) (Table 1).

AT elasticity decreased significantly from baseline (1.75 ± 0.032) to post exercise conditions 1.39 ± 0.036 (*p* < 0.000) (Table 1).

Average AT thickness at baseline was 8.87 ± 0.54 mm and 6.80 ± 0.74 mm after activation with the half squat exercise (*p* < 0.000) (Table 1).

## 4. Discussion

This study assessed the impact of post activation potentiation on Achilles tendon (AT) stiffness and thickness among basketball players. To the best of our knowledge, this is the first study to compare Achilles tendon stiffness, elasticity and thickness after PAP. Therefore, the results of this study will provide relevant information to medical staff, athletes, and coaches.

In our research we found that AT thickness decreased significantly after resistance exercises. This is in line with other studies. According to Tardioli et al. major evidence that tendon diameter decreases directly after physical effort, even when the meaning of this adaptive mechanism is unknown [25]. Fahlstrom and Alfredson found in their research that one hour of high-intensity exercise decreases AT diameter [26]. According to Grigg, eccentric calf muscle exercise produces a greater acute reduction in AT thickness than concentric exercise [27]. Van Drongelen et al. examined the correlation between practice time and increased biceps tendon diameter [28]. They assumed that the increasing time of the training might play a significant role in tendon thickness changes [28].

Our research indicates that AT stiffness increased after resistance exercise. It can be the effect of multiple tensile strain cycles that the AT undergoes during the movements. Wu et al. [29] identified that the increase in tendon stiffness as a result of a plyometric training program was related to significant improvement in countermovement jump height. On the other hand, Kubo et al. found that plyometric training increased joint stiffness yet not tendon stiffness, whilst weight training increased tendon stiffness but not joint stiffness [30]. Kalkhoven in his study suggested that the tendon is the main modulator of force velocity and force length relationship [31]. Alexander and Bennet-Clark [32] have shown that the wallaby’s AT can store up to ten times as much energy as the muscle [32]. Tendons are like buffers. Roberts et al. [33] reported that under circumstances that require the dissipation of mechanical energy, the elastic behavior of the tendon can protect the muscle by temporarily storing energy rapidly, then releasing it more slowly to work on the muscle. This mechanism may protect muscles from damage by reducing the rate of muscle fascicle lengthening and peak power inputs to muscle fascicles [33]. We found that after exercises that trigger the PAP effect, stiffness increased. Accordingly, as stiffness increases, less activation is required, and therefore energy expenditure is spared. This could be useful information for coaches. It has to be underlined that there is no research about the influence of PAP on AT viscoelastic properties, especially in basketball players. The differences between our study and the studies that we refer to are likely methodological. Most of the other research used ultrasonography as a measurement method.

The results of our study can be an important indication for people concerned with Achilles tendinopathy. Obst et al. in their research found that people with Achilles tendinopathy had a lower AT global stiffness, lower global modulus and lower local modulus, compared to asymptomatic controls [34]. Results of this study strongly support the need of heavy weight training as a preventing measure in endurance runners or other athletes.

Collagen is one of the main components that is responsible for maintaining cohesiveness, tensile strength and tendon elasticity. In our research we found that AT elasticity decreased after the PAP session. This may be caused by a reduction in the level of collagen after physical effort. Landberg et al. [35] propose that collagen degradation occurs right after exercise and is followed by a significant growth in collagen turnover for up to 72 h after physical effort. Other scholars also confirmed an initial decrease in Achilles collagen turnover after exercise [36,37]. Tardioli showed that the peak of the collagen degradation happened earlier (36 h) than collagen synthesis (up to 72 h). The crucial finding presumes that elite athletes during repeated and high-intensity training require particular recovery programs for optimal net anabolic collagen turnover [25]. This theory is only speculative because we did not measure collagen turnover.

The extracellular matrix (ECM) of the tendon is composed predominantly of collagen, which accounts for ~60–85% of the dry weight of the tissue [38]. Current knowledge of tendon remodeling mechanisms is incomplete and inconsistent but there are leads that components of the extracellular matrix (ECM) play crucial roles in this process [39]. Proteoglycans (PGs) are particularly prominent in the pericellular regions, but also in compressive regions of tendons [40,41]. Their role, (increasing water content in these regions), provides resistance to compression [42]. One of the interfibrillar sulfated proteoglycans is decorin which is named because of its ability to decorate collagen fibrils, decreases mechanical properties and corresponds to about 90% of all tendon PG content [43,44,45]. Yoon and Halper in their research found that electron microscopic examinations of the skin in decorin-knockout mice show that in the absence of decorin, collagen fibrils are coarse, irregular and haphazardly arranged [45]. These changes are accompanied by a decrease in collagen-bound PGs in the skin and tendon. Franchi et al. in their research observed a decorin increase in running animals. They suggested that decorin may play an important role in retaining water in the inter-fibril spaces, and might oppose fibril shrinkage and lubricate fibril surface, favoring fibril sliding [46]. In our opinion, further studies are needed to develop the whole issue completely.

The present study provides important information about AT adaptation mechanisms in response to PAP. Additional research on athletes from other disciplines is needed, but strength and conditioning professionals should consider our results in designing training programs, especially those that include PAP and other strength training methods [47,48].

## Figures and Tables

**Table 1:** Achilles Tendon stiffness, elasticity and thickness before and after PAP session.

Session	Achilles Tendon Stiffness (N/m)	Achilles Tendon Elasticity	Achilles Tendon Thickness (mm)
Before PAP	727.42 ± 111.57	1.75 ± 0.032	8.87 ± 0.54
After PAP	822.50 ± 119.55	1.39 ± 0.036	6.80 ± 0.74
